# Optimizing the clinical utility of PCA3 to diagnose prostate cancer in initial prostate biopsy

**DOI:** 10.1186/s12885-015-1623-0

**Published:** 2015-09-11

**Authors:** Jose Rubio-Briones, Angel Borque, Luis M. Esteban, Juan Casanova, Antonio Fernandez-Serra, Luis Rubio, Irene Casanova-Salas, Gerardo Sanz, Jose Domínguez-Escrig, Argimiro Collado, Alvaro Gómez-Ferrer, Inmaculada Iborra, Miguel Ramírez-Backhaus, Francisco Martínez, Ana Calatrava, Jose A. Lopez-Guerrero

**Affiliations:** 1 Department of Urology, Instituto Valenciano de Oncología, C/ Prof. Beltrán Báguena 8, 46009 Valencia, Spain; 2 Department of Urology, Hospital Universitario Miguel Servet, Zaragoza, Spain; 3 Escuela Universitaria Politécnica de La Almunia, Universidad de Zaragoza, Zaragoza, Spain; 4 Laboratory of Molecular Biology, Instituto Valenciano de Oncología, Valencia, Spain; 5 Department of Statistical Methods, Universidad de Zaragoza, Zaragoza, Spain; 6 Department of Statistics, University of Valencia, Valencia, Spain; 7 Department of Pathology, Instituto Valenciano de Oncología, Valencia, Spain

## Abstract

**Background:**

PCA3 has been included in a nomogram outperforming previous clinical models for the prediction of any prostate cancer (PCa) and high grade PCa (HGPCa) at the initial prostate biopsy (IBx). Our objective is to validate such IBx-specific PCA3-based nomogram. We also aim to optimize the use of this nomogram in clinical practice through the definition of risk groups.

**Methods:**

*Independent external validation*. Clinical and biopsy data from a contemporary cohort of 401 men with the same inclusion criteria to those used to build up the reference’s nomogram in IBx. The predictive value of the nomogram was assessed by means of calibration curves and discrimination ability through the area under the curve (AUC). Clinical utility of the nomogram was analyzed by choosing thresholds points that minimize the overlapping between probability density functions (PDF) in PCa and no PCa and HGPCa and no HGPCa groups, and net benefit was assessed by decision curves.

**Results:**

We detect 28 % of PCa and 11 % of HGPCa in IBx, contrasting to the 46 and 20 % at the reference series. Due to this, there is an overestimation of the nomogram probabilities shown in the calibration curve for PCa. The AUC values are 0.736 for PCa (C.I.95 %:0.68–0.79) and 0.786 for HGPCa (C.I.95 %:0.71–0.87) showing an adequate discrimination ability. PDF show differences in the distributions of nomogram probabilities in PCa and not PCa patient groups. A minimization of the overlapping between these curves confirms the threshold probability of harboring PCa >30 % proposed by Hansen is useful to indicate a IBx, but a cut-off > 40 % could be better in series of opportunistic screening like ours. Similar results appear in HGPCa analysis. The decision curve also shows a net benefit of 6.31 % for the threshold probability of 40 %.

**Conclusions:**

PCA3 is an useful tool to select patients for IBx. Patients with a calculated probability of having PCa over 40 % should be counseled to undergo an IBx if opportunistic screening is required.

## Background

Urologists need tools to optimize the performance of an initial prostate biopsy (IBx) as this procedure is related to emotional stress derived from a potential cancer diagnoses [1] and adverse biopsy-related events such as bleeding, urinary obstructions and infections [2, 3].

PCA3 as a single biomarker has been approved by the FDA to guide prostatic biopsy (Bx) in men with a negative previous IBx. On the other hand, we and others [4–7] have reported better results on patients not previously biopsied.

Nomograms help clinicians to estimate the probabilities associated in different scenarios of the disease and are essential for counseling patients [8–11]. PCA3 has been included in nomograms to predict prostate cancer (PCa) at IBx or repeated Bx [5, 12–14]. In this paper we focus our attention into a recently published nomogram by Hansen et al. that also studied PCA3 as a marker for the prediction of any PCa at the IBx and its ability to identify high-grade PCa (HG-PCa; considered as Gleason score at biopsy ≥ 7). These authors concluded that the addition of PCA3 to a set of standard risk factors improves significantly the discrimination ability of a predictive model of PCa, avoiding unnecessary IBx [15].

Our aim is to externally validate such IBx-specific PCA3-based nomogram in a single center cohort and to optimize its use in clinical practice through the definition of risk groups. We used a graphical procedure to establish a threshold point for this nomogram through the use of probability density functions (PDF) of harboring or not PCa, favoring its implementation for clinical use [16].

## Methods

### Patient population

We enrolled 613 men scheduled for IBx with PCA3 testing in our daily practice. Selection criteria were the same as in the Hansen’s cohort [15] that was built with 692 patients from two prospective multi-institutional studies in Europe [4] and USA [17] with suspicious DRE or PSA between 2.5 and 10 ng/ml and a minimum of 10 cores IBx. In case of suspicious DRE, men with PSA between 10 and 20 ng/ml were included. Prostate volume was determined by ultrasound and urine infection ruled out. Finally, from the whole series 401 men referred to IBx met all the established selection criteria. This study was approved by the Ethics Committee of the Fundación Instituto Valenciano de Oncología (ref. number. 2010–20). At the moment of the urine collection for the PCA3 analysis all patients gave their consent for the use of the leftover urine and associated information for research purposes following the standards set by the Institutional Biobank (Spanish Biobank Registry number: B.0000773; https://biobancos.isciii.es/ListadoBiobancos.aspx?id=B.0000773).

### Clinical evaluation

PCA3 was performed following manufacturer’s instructions [18] and DRE was reported as unsuspicious versus suspicious. Transrectal ultrasound (TRUS)-derived total prostate volume was calculated using the prostate ellipse formula (0.52 × length × width × height). 10–12 core systematic laterally directed TRUS guided biopsies were performed. All biopsy specimens were evaluated by a single experienced uropathologist (AC).

### Statistical analysis

The external validation was performed analyzing the calibration, discrimination and clinical utility [10, 19]. The calibration is analyzed by means of calibration curves and the two informative parameters: Intercept (calibration-in-the-large) and Slope, which evaluate the correspondence between the predicted and the actual probabilities. To study the discrimination ability and the clinical utility of the model, the empirical distributions of probabilities of PCa in the PCa/No-PCa and HGPCa/No-HGPCa populations have been estimated. Those probabilities are estimated in the IVO cohort using the Hansen nomogram by kernel density estimation [20]. The way in which the probability distributions of PCa populations overlap is important to know how the model discriminates between groups and to show the best threshold to define risk groups for clinical use. Moreover, discrimination has been quantified through the Receiver Operating Characteristics (ROC) curve [21], the area under the ROC curve (AUC) and its 95 % confidence interval (CI). We also evaluate its clinical utility through Vickers’ decision curves [22] that analyze the net benefit for different threshold probabilities. Statistical analyses were performed using R programming language v.3.1.0 [23].

## Results

Table 1 summarizes the characteristics of patients of the two multi-institutional studies included in the Hansen study and the IVO cohort. Our PCa detection rates were 28 % (11 % HGPCa), clearly lower than the Hansen’s series values of 46 % (20 % HGPCa). The median age was 2 years lower in the IVO cohort, but interquartile ranges (IQR) were very similar. Prostate volume results were quite similar for the both cohorts, but importantly the Wilcoxon signed rank test p-value <0.001 confirmed differences with regard median PSA levels (5.2-Hansen vs 4.3-IVO ng/ml). Also, differences were observed in the percentage of patients with suspicious DRE (28.5 vs 10.7; *p* < 0.001) and with suspicious DRE and PSA between 10 and 20 ng/ml (4 vs 1 %; *p* < 0.01).

**Table 1:**
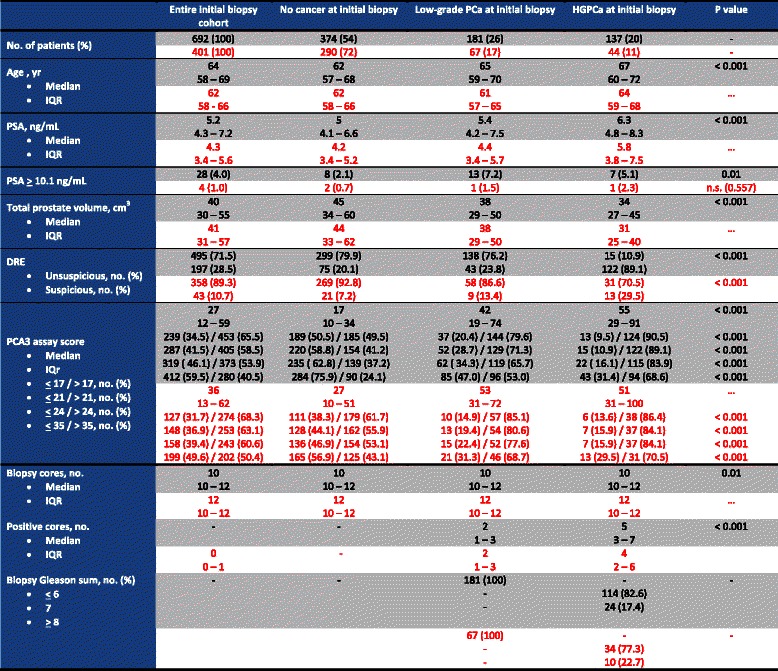
Data from men included in the referenced nomogram (in black) and from the IVO series (in red)

Median PCA3 values were 27 and 36 for Hansen and IVO series respectively. The Wilcoxon signed rank test showed that PCA3 median value in the IVO cohort (*p* < 0.001) showed a statistically significant difference with the Hansen median value. Attending to the manufacturer threshold of 35, we observed 50.4 % of the IVO series with PCA3 values above 35 and just 40.5 % for the reference series. The test for equality of proportions had a p-value < 0.001, therefore showing again statistically significant differences between these cohorts. We observed decreasing sensibilities from lower to higher PCA3 cut-offs for PCa detection in similar percentages of both series, showing statistically significant differences in all thresholds when compared to negative IBx.

Furthermore, Table 2 shows the univariant association and the discrimination power measured by the AUC for each predictor variable in the diagnosis of PCa and HGPCa. Continuous PCA3 variable has the maximum AUC value of 0.701 for PCa diagnosis in our series. Using the De Long test for comparison of AUC between PCA3 and the rest of predictors, statistically significant differences were established in all cases except for the comparison with the prostatic volume.

**Table 2:**
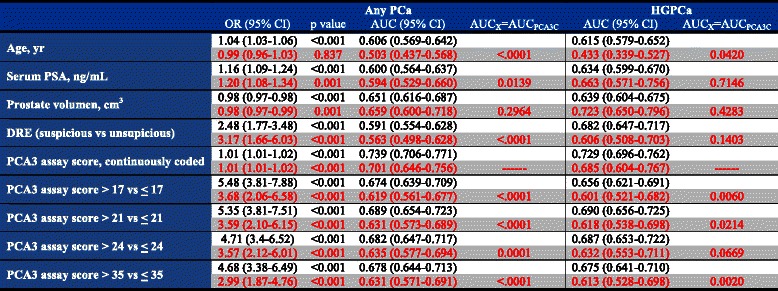
Univariant analyses and AUC for each variable for the detection of any PCa and HGPCa in Hansen’s series (black) and IVO series (red)

**Fig. 1:**
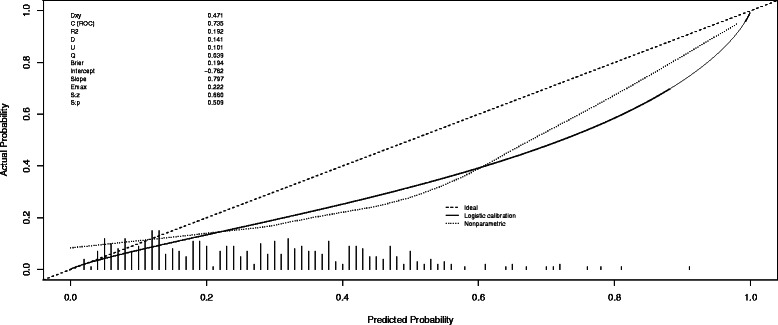
Calibration curve applying Hansen’s nomogram to the IVO’s series, for the detection of any PCa

In Fig. 1 we show the calibration curve. There is an important overestimation in all range of probabilities for the detection of any PCa, due to the difference in the prevalence of PCa between the Hansen and the IVO cohorts. The intercept and Slope values of −0.762 and 0.797 confirm a poor calibration.

**Fig. 2:**
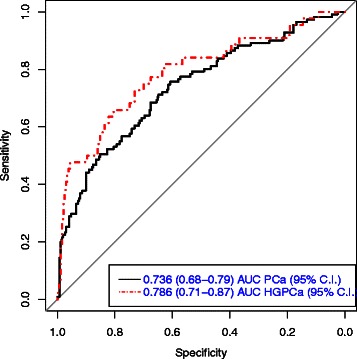
Area under the curve for the detection of any PCa and for the detection of HGPCa

**Fig. 3:**
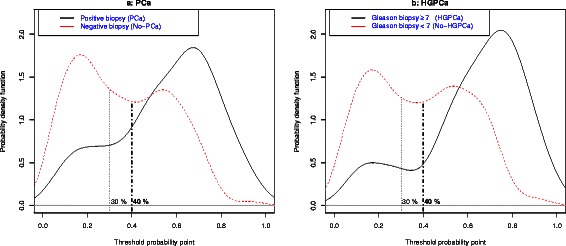
**a** Probability distribution of any PCa detection through Hansen’s nomogram; **b** probability distribution of HGPCa detection through Hansen’s nomogram

Regarding the discrimination ability of the nomogram, we obtained an AUC for the detection of any PCa of 0.736 (CI 95 %: 0.680–0.793) and a value of 0.786 (CI 95 %: 0.705–0.867) for the detection of HGPCa (Fig. 2). The distribution of probabilities of PCa assigned by the nomogram to our patients with and without real PCa are shown in Fig. 3a, and for HGPCa/Non HGPCa in Fig. 3b. These density functions curves show than 40 % is the best cut off better pointing to the probability of harboring any PCa above it (Fig. 3a), same value when focusing on HGPCa (Fig. 3b).

Therefore, facing the decision of indicating an IBx, we propose 40 % as a better cut off than the 30 % Hansen et al. proposed for their nomogram. A higher number of patients without PCa and particularly without HGPCa are correctly classified under the threshold point of 40 % of the nomogram, with a very scarce number of patients with PCa and HGPCa missed in the interval between 30 and 40 % (Fig. 3a and b).

**Fig. 4:**
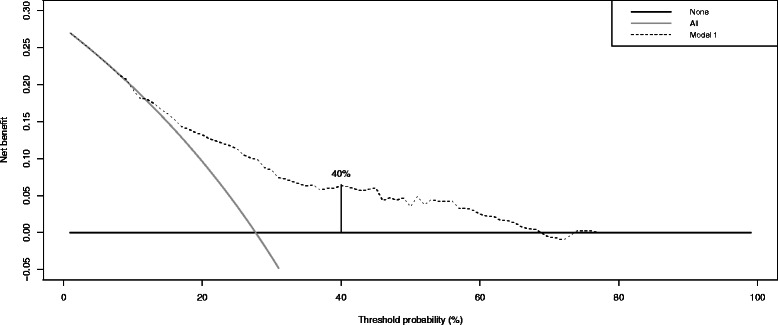
Net benefit curves following Vickers’ decision curves

Finally, Vickers’ decision curves show the net benefit obtained from the application of the Hansen’s nomogram to the entire IVO-cohort. We check the improvement provided by the model for different cut-off probabilities (Fig. 4).

## Discussion

Several nomograms optimize the indication of IBx, with predictive accuracy estimates between 67 and 77 % [3, 24–26]. In particular, the PCA3-based nomogram validated here showed that the accuracy of the clinical model was increased by 4.5–7.1 % related to PCA3 inclusion [15]. In this model, continuously coded PCA3 represented the most informative parameter in the prediction of any PCa (AUC = 0.739) and HGPCa (AUC = 0.729). In our univariate analyses, we also showed that continuous coded PCA3 was the best predictive variable for the detection of any PCa (AUC = 0.701) and prostate volume was slightly better to detect HGPCa (AUC = 0.723) than continued coded PCA3 (AUC = 0.685). Checking different cut-offs, 24 and not 21, showed the highest AUC for both any PCa and HGPCa (Table 2). These findings agree with previous studies [27]. We also tested categorized PCA3, and we agree with the Hansen et al. series that continuous coded PCA3 was the most informative variable to predict PCa and HGPCa, therefore it should be considered this way when building a nomogram. The non-linear effects of PCA3 can be appropriately modeled using its continuous form, thus adding more predictive power [28].

The calibration plot and the values of intercept and slope showed a poor agreement between actual and predicted probabilities obtained from the application of the nomogram to our 401 men as a validation cohort (Fig. 1). This fact is explained by the clear differences in detection rates between both series. Our PCa detection rate was 28 % (11 % HGPCa), closer to the expected prevalence of PCa in contemporary series of opportunistic screening, and the same features were 46 % (20 % HGPCa) in the referenced series. The initial cohorts which generated Hansen’s nomogram consisted in 570 patients enrolled from 4 North American sites with a detection rate of PCa of 36 % (15 % HGPCa) [17] and 516 patients from a European multicenter study with a detection rate of PCa of 40 % (19 % HGPCa) [4], closer to our data. From the entire cohort of 1086 patients, 692 patients were finally considered to generate Hansen’s nomogram and the proportion of PCa and HGPCa increased substantially (46 % PCa and 20 % HGPCa) from original cohorts. We think this fact could explain their high prevalence of PCa and HGPCa, which overcomes expected rates of PCa and HGPCa for patients with PSA between 2,5 and 10 ng/mL. In this sense we think our detection rates are more similar to the reported in the literature, with most men coming from opportunistic screening scenarios. Only series with so extremely high rate of PCa/HGPCa could expect a nice calibration plot. In any case, a good calibration would had shown a good performance of the Hansen et al. model in our series, but a bad calibration doesn’t mean that the validated nomogram is a bad predictive model in our series, but just a different threshold point must be investigated.

We obtained slightly inferior AUC (0.736, CI95 %:0.680–0.793) for any PCa detection that the referenced nomogram (AUC 0.807, CI95 %:0.768–0.828), showing statistically significant differences between AUCs (p-value = 0.02), but similar to the other published external validation (0.764, CI95 %:0.726–0.802) [27], p-value = 0.43, so we think the model offers good discrimination ability. When we built a logistic regression model using the same predictive variables and using PCA3 as a continuous value we obtain an AUC of 0.769 (data not shown), very similar to the application of Hansen nomogram in our series (Table 3). Recently, a similar evaluation of the use of PCA3 as a continuous predictor in a multivariate logistic regression model developed over 3073 patients from screening population in USA, showed an AUC of 0.75 and 0.81 respectively for the prediction of PCa and HGPCa respectively at the IBx [29].

**Table 3:** Logistic regression model using the same predictive variables and using PCA3 as a continuous value

	Clinical variables + PCA3		Clinical variables	
Predictor variable	O.R. (95 % C.I.)	p-value	O.R. (95 % C.I.)	p-value
PSA	1.63 (1.21–2.20)	0,001	1.69 (1.27–2.26)	<,001
Prostatic volume	0.54 (0.38–0.77)	<,001	0.45 (0.32–0.63)	<,001
DRE	3.57 (1.67–7.61)	0,001	3.64 (1.76–7.53)	<,001
PCA3	3.19 (2.08–4.88)	<,001		
AUC	0.769 (0.72–0.82)		0.712 (0.66–0.77)	
AUC comparison	p-value = 0.008			

In the decision curve analysis we obtained at a 30 % threshold probability a net benefit 8.41 %, superior to the baseline model, but far away of the >18 % recognized for the model in the original series. For a threshold probability of 40 % the net benefit obtained is 6.31 %. Other authors have also shown that decision curve analysis confirmed a higher benefit when adding the PCA3 score (either continuous or binary with a cutoff of 35) to the baseline model [27] in IBx. In the logistic regression model built with our database using the continuous PCA3 score, the net benefit is 6.90 % for the 40 % threshold point, very similar to the Hansen nomogram application.

Multivariate models translate multiple effects in one number, which is the interpretation of the risk of harboring an event from 0 to 100 %. But we as clinicians take decisions based on a reference value (PSA > 4 ng/ml, free-PSA ratio < 15 %, etc.), counseling patients taking based on them. With the aim of helping the clinician to indicate or not an IBx, we investigated the probabilities of the model to detect PCa through PDF [16]. These density functions help us to choose thresholds to differentiate groups of high and low risk probabilities of harboring PCa (Fig. 3a) or HGPCa (Fig. 3b). The density curves of probabilities are built from the probabilities of having PCa assigned by the nomogram to each man. In their X-axis, we reflect the range of possible probabilities (0–100) of harboring PCa according to the nomogram. In the Y-axis we range the density of patients from their assigned probability of harboring PCa or HGPCa. We can see the higher peak of density in PCa patients near the X = 70 % probability of harboring PCa in Fig. 3a and in HGPCa patients near 80 % in Fig. 3b. On the other hand, this peak appears around X = 15–20 % for non PCa/non HGPCa patients.

We can easily see how our PDF curves show a “valley” between the “peak” of patients with PCa/HGPCa in the range of high probabilities of harboring PCa (in the right side of the graphic), and the “peak” of patients without PCa/HGPCa in the range of low probabilities (in the left side). This valley drives us to choose the threshold of probability to classify patients in high or low risk of harboring PCa. A threshold point of 40 % instead of 30 %, as proposed by Hansen et al., could be the best option to translate the implementation of this nomogram in our daily practice.

Applying the nomogram with a threshold of 40 % to our 401 men, we would had saved 197 IBx (49.1 %), at a cost of missing 27 any PCa (24.3 %) and 7 HGPCa (15.9 %) (Table 4). For the threshold value of 30 % provided by Hansen et al., we would had saved 151 IBx (37.3 %) at a cost of missing 21 any PCa (19.9 %), and 6 HGPCa (13.4 %). Therefore, selecting 40 %, nor 30 %, we would had saved 11.8 % IBx more just missing one HGPCa more. This features would always improve the results of taking single PCA3 cut offs values as a single tool to decide IBx (Table 5), where we can check that if we had chosen PCA3 > 21 we would had missed 15.9 % HGPCa, similar to the 13 % observed by other authors at IBx with a cut-off of 20 [30], but doing 12.2 % more IBx that if we had applied the nomogram. We notice that our small number of HGPCa (44 cases) could affect our data on this population, as using the threshold point of 40 % the percentage of missed HGPCa cases is 15.9 %, but the 95 % CI is 7.1–30.7 %.

**Table 4:** Potential avoided initial biopsies (IBx), PCa and HGPCa detection and missed rates at IBx using different threshold probabilities values

Threshold	Biopsies	Biopsies	PCa detected	PCa delayed	HGPCa detected	HGPCa delayed
Probability	Perforrmed, n (%)	Avoided, n (%)	n (%)	Diagnosis, n (%)	n (%)	Diagnosis, n (%)
>10 %	362 (90.3 %)	39 (9.7 %)	108 (97.3 %)	3 ( 2.7 %)	43 (97.7 %)	1 ( 2.3 %)
>20 %	295 (73.6 %)	106 (26.4 %)	99 (89.2 %)	12 (10.8 %)	40 (90.9 %)	4 ( 9.1 %)
>30 %	250 (62.3 %)	151 (37.3 %)	90 (81.1 %)	21 (19.9 %)	38 (86.4 %)	6 (13.4 %)
>35 %	228 (56.9 %)	173 (43.1 %)	88 (79.3 %)	23 (20.7 %)	37 (84.1 %)	7 (15.9 %)
>40 %	204 (50.9 %)	197 (49.1 %)	84 (75.7 %)	27 (24.3 %)	37 (84.1 %)	7 (15.9 %)
>45 %	187 (46.6 %)	214 (53.4 %)	80 (72.1 %)	31 (27.9 %)	36 (81.8 %)	8 (18.2 %)
>50 %	160 (39.9 %)	241 (60.1 %)	71 (64.0 %)	40 (36.0 %)	34 (77.3 %)	10 (22.7 %)
>60 %	98 (24.4 %)	303 (75.6 %)	55 (49.5 %)	56 (50.5 %)	28 (63.6 %)	16 (36.4 %)

**Table 5:** Potential avoided initial biopsies (IBx), PCa and HGPCa detection and missed rates at IBx using various threshold PCA3 values as a single decision tool

Threshold	Performed biopsies	Avoided biopsies	PCa detected	PCa delayed	HGPCa detected	HGPCa delayed
Probability	n (%)	n (%)	n (%)	Diagnosis n (%)	n (%)	Diagnosis n (%)
PCA3 > 17	280 (69,8)	121 (30,2)	96 (86,5)	15 (13,5)	38 (86,4)	6 (13,6)
PCA3 > 21	256 (63,8)	145 (36,2)	93 (83,8)	18 (16,2)	38 (86,4)	6 (13,6)
PCA3 > 24	245 (61,1)	156 (38,9)	90 (81,1)	21 (18,9)	37 (84,1)	7 (15,9)
PCA3 > 25	243 (60,6)	158 (39,4)	89 (80,2)	22 (19,8)	37 (84,1)	7 (15,9)
PCA3 > 35	206 (51,4)	195 (48,6)	78 (70,3)	33 (29,7)	32 (72,7)	12 (27,2)

It would had been desirable to compare the initial clinical nomogram built without PCA3 evaluated by Hansen et al. to ours, in order to know the clinical benefit of determining PCA3, but that nomogram was not published. We show in Table 3 that there are statistically significant differences (*p* < 0.01) between models build with or without PCA3 as predictor variable.

From a practical point of view, and in order to save costs, we ask for PCA3 just in doubtful cases, in the way Abern and Freedland propose [31]. If we had applied the nomogram to our series, we would had obtained a score of 121 total points (equivalents to a probability of ≥ 40 %) in 204 men. Twenty-six of them would had had 121 points without the need to test PCA3, so we would had indicated the IBx saving costs. In the lower scenario, 178 would not had reached to 121 points adding the additional 26 points dependent on a PCA3 > 21, but we think that not using the aid of PCA3 at this scenario, knowing the strength of PCA3 as a continuous variable and that prostate volume could be undermeasured by hypogastric sonography, that the cost of PCA3 would be worth while for a better counseling of IBx to a men in this grey area.

Finally, this external validation in a single center over a series of 401 patients is closer to a opportunistic screening scenario with a prevalence of PCa of 28 %, more common than the 46 % given by the referenced nomogram. This fact makes it particularly applicable in daily practice compared to the referenced nomogram (international, multicentre, multiethnic, different PSA assays used).

## Conclusions

We validate the PCA3-based nomogram in IBx published by Hansen et al. reinforcing its higher utility when PCA3 is used within a nomogram and selecting cases for its use. We find an overestimation of probabilities and minimal loss in the discrimination power of the model, but we can confirm it as a valid tool for our population. Using a new methodology, we propose 40 % as the most reliable threshold point to use the proposed nomogram recommending or not a healthy man an IBx in front of an opportunistic screening. This threshold offers us an optimal tool to help a well-informed man in his decision.

## Abbreviations

AUC: Area under the curve
Bx: Prostatic biopsy
CI: Confidence interval
FDA: Food and drug administration
DRE: Digital rectal examination
HG-PCa: High-grade PCa
IBx: Initial prostate biopsy
IVO: Instituto Valenciano de Oncología
IQR: Interquartile ranges
PCa: Prostate cancer
PCA3: The Prostate CAncer gene 3
PDF: Probability density functions
PSA: Prostatic specific antigen
TRUS: Transrectal ultrasound
ROC: Receiver operating characteristics
USA: United States of America
